# Multi-omics analysis of cecal microbiota-hypothalamus axis interactions in small-sized meat ducks with divergent residual feed intake

**DOI:** 10.1016/j.psj.2026.107310

**Published:** 2026-06-17

**Authors:** Dandan Geng, Yifan Ding, Yong Jiang, Zhixiu Wang, Guohong Chen, Guobin Chang, Hao Bai

**Affiliations:** a Joint International Research Laboratory of Agriculture and Agri-Product Safety, the Ministry of Education of China, Institutes of Agricultural Science and Technology Development, Yangzhou University, Yangzhou 225009, China; b Key Laboratory of Animal Genetics and Breeding and Molecular Design of Jiangsu Province, Yangzhou University, Yangzhou 225009, China

**Keywords:** Residual feed intake, Small-sized meat ducks, Hypothalamus, Cecal microbiota, Gut-brain axis

## Abstract

Residual feed intake (**RFI**) is an indicator of feed efficiency that reflects variation in nutrient utilization independent of growth. This study characterized physiological traits and multi-omics profiles associated with divergent RFI in small-sized meat ducks. From an initial population of 500 1-day-old ducks, a total of 420 healthy ducks were individually housed from 21 to 42 d to record feed intake, and ducks with low RFI (**LRFI**) and high RFI (**HRFI**) were identified for further analyses. During the experiment, 30 ducks per group for growth performance, 15 ducks per group for plasma biochemical and 5 per group for multi-omics. Compared with HRFI ducks, LRFI ducks showed lower feed intake, lower feed conversion ratio (**FCR**), and lower plasma triglyceride concentrations, whereas body weight gain did not differ between groups. Shotgun metagenomic analysis showed that LRFI ducks were enriched in *Bacteroides*-related lineages and had higher predicted capacities for complex carbohydrate degradation, lipid and energy metabolism, and cofactor synthesis, whereas HRFI ducks were enriched in taxa including *Subdoligranulum variabile* and *Clostridioides difficile*. Untargeted cecal metabolomics revealed distinct lipid- and bile acid-related metabolic profiles between the 2 groups, including differences in long-chain lipid species and bile acid-associated metabolites. Hypothalamic transcriptomic analysis identified differentially expressed genes related to neuropeptide signaling, serotonin biosynthesis, intracellular signaling, and inflammatory regulation, including *NMUR2, TPH1*, and *PTK2B*. Correlation analysis integrating microbial taxa, metabolites, and hypothalamic transcripts further revealed coordinated associations among these features in small-sized meat ducks with divergent RFI. Overall, variation in feed efficiency in ducks was associated with differences in cecal microbiota, metabolite profiles, and hypothalamic gene expression, and these results highlight candidate microbial taxa, metabolites, and genes for further validation.

## Introduction

As global demand for meat continues to increase, duck meat has become an important source of animal protein. China is a major producer and consumer of meat ducks. In 2024, the national output of waterfowl exceeded 5.0 billion birds, including approximately 4.22 billion commercial meat ducks (Hou and Liu, 2026). Small-sized meat ducks are favored by consumers because of their compact body size, strong stress tolerance, and desirable meat quality. However, their relatively slow growth and poor feed efficiency remain major constraints to industrial production. Residual feed intake (**RFI**) is widely used to evaluate individual feed efficiency and is defined as the difference between observed feed intake and the intake expected for maintenance and growth (Koch et al., 1963). Previous studies have shown that selection for low RFI can improve feed efficiency without adversely affecting slaughter performance or meat quality (Do, et al., 2026; Gurgeira, et al., 2022; Wu, et al., 2025). In poultry, RFI has also been reported to be associated with carcass composition and muscle development traits (Chen, et al., 2025b; Wan, et al., 2025), suggesting potential links with energy metabolism and related tissue functions. In recent years, poultry RFI studies have increasingly moved beyond performance traits and have focused on the biological mechanisms underlying feed efficiency. Studies in chickens and ducks have indicated that divergent RFI phenotypes are associated with changes in nutrient digestion (Li, et al., 2023), intestinal microbial composition (Bai, et al., 2022b; Yang, et al., 2024), host metabolism (Qin, et al., 2023), immune-related responses, and gene expression profiles. In ducks, recent microbiome- and metabolome-based studies have further suggested that intestinal microbial communities and microbial-derived metabolites may contribute to individual differences in feed utilization efficiency (Sun, et al., 2023). However, the biological factors associated with RFI variation in ducks remain poorly understood. A better understanding of these factors may provide useful information for future breeding and nutritional strategies aimed at improving feed efficiency in commercial duck production.

To investigate the biological basis of variation in RFI, it is important to understand how nutrient utilization in the gut and appetite regulation in the brain may be coordinated. The gut–brain axis provides a useful framework for examining these relationships, in which intestinal microbial activity generates metabolic and endocrine signals that may be sensed by hypothalamic centers involved in feeding and energy balance. In poultry, the paired ceca are major sites of microbial fermentation and nutrient metabolism (Montso, et al., 2022). Shifts in cecal community structure and functional capacity have been associated with changes in the breakdown and availability of dietary substrates and with variation in feed efficiency (Wen, et al., 2021; Yang, Dong, Chen, Jiang, Bai, Chen, Chang and Wang, 2024). Cecal microbes may shape host energy utilization through metabolites such as short-chain fatty acids and vitamins, and these metabolites have been reported to engage host immune and neuroendocrine signaling pathways (Liu, et al., 2024; Yang, et al., 2022). Consistent with this concept, previous duck RFI studies combining intestinal microbiota and metabolomics have identified differential microbial taxa, metabolites, and metabolic pathways related to amino acid metabolism, vitamin metabolism, lipid metabolism, and membrane transport. These findings indicate that metabolic regulation may be an important link between gut microbial activity and feed efficiency variation. In the hypothalamus, hormonal, inflammatory, and nutrient-related signals are integrated to regulate feeding behavior and energy balance, and changes in hypothalamic gene expression have been associated with shifts in these signaling (D'Alessio, et al., 2026). Classic neuroendocrine regulators, including neuropeptide Y (**NPY**) and proopiomelanocortin (**POMC**), have been implicated in feed intake regulation in poultry. Together, these observations are consistent with a gut–brain axis model in which cecal microbial activity may be associated with hypothalamic transcriptional profiles and feeding behavior relevant to RFI. In ducks, previous work combining cecal 16S rRNA profiling with hypothalamic RNA sequencing reported correlations between specific microbial taxa and hypothalamic gene expression in birds differing in feed efficiency (Bai, et al., 2024). However, that study did not include untargeted metabolomics or shotgun metagenomics. As a result, it remains unclear whether specific cecal taxa are associated with particular metabolites that are in turn related to hypothalamic gene expression differences in high- versus low-RFI ducks. Therefore, an integrated multi-omics approach is needed to place cecal microbial composition, microbial functional potential, metabolic regulation, and hypothalamic transcriptional responses into a unified framework for understanding RFI variation in ducks.

To address this gap, the present study profiled the cecal microbiome by shotgun metagenomics, the cecal metabolome by untargeted LC-MS, and the hypothalamic transcriptome by RNA sequencing in small-sized meat ducks with divergent RFI. Through multi-omics association analyses, we examined the relationships among microbial taxa, their functional pathways, cecal metabolites, and hypothalamic gene expression, and identified candidate features associated with feed efficiency variation. These findings may provide useful information for understanding microbiota-metabolite-hypothalamus interactions associated with RFI and for future studies aimed at improving feed efficiency in commercial small-sized meat ducks.

## Materials and methods

### Ethics statement

All experimental procedures involving animals were approved by the Animal Care and Use Committee of Yangzhou University (Protocol No. SYDW-2019015) and were conducted in accordance with the committee's guidelines for the care and use of animals in research.

### Animal preparation and experimental design

**Fig. 1:**
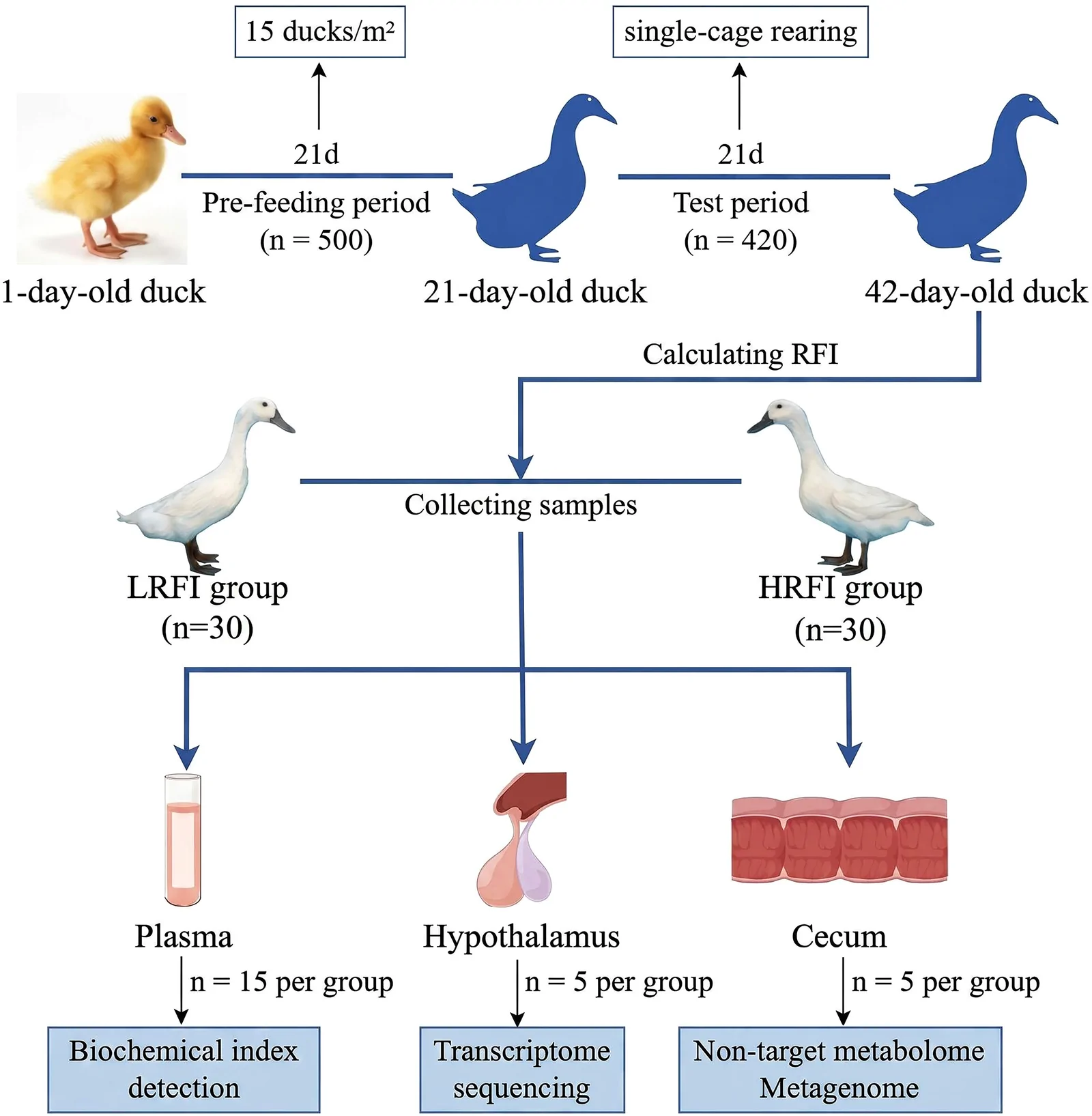
Overview of the experimental design. A total of 500 mixed-sex ducklings were initially used, and 420 ducks entered the individual feeding trial. After RFI calculation, 30 ducks with the lowest RFI values and 30 ducks with the highest RFI values were selected as the LRFI and HRFI groups, respectively, for growth performance analysis. Among them, 15 ducks per group were used for plasma biochemical analysis, and 5 ducks per group were used for metagenomic, metabolomic, and transcriptomic analyses. Cecal contents and hypothalamic tissues used for multi-omics analyses were collected from the same 5 individual ducks per group.

The experiment was conducted using the H1 strain of small-sized white-feathered meat ducks, a commercial line maintained at the Special Duck Breeding Farm of Shuyang Zhongke Poultry Co., Ltd. (Jiangsu, China). A total of 500 mixed-sex ducklings were obtained at hatch and reared as a single cohort. From hatch to 21 d of age, birds were reared in floor pens at a density of 15 birds/m² and fed a standard basal diet with ad libitum access to water. Temperature and lighting followed the routine management protocol for this line. At 21 d of age, birds were screened for health and body weight. Birds with clinical illness, injury, or growth retardation were excluded, and 420 ducks with comparable body weights were selected and housed individually in single cages (73 × 55 × 80 cm). The individual test phase lasted from 21 to 42 d of age, during which each duck had ad libitum access to feed and water. During this phase, all ducks were kept in the same house under identical management conditions, with feed supplied at the same time each day and temperature, ventilation, and lighting maintained consistently across cages. The basal diet formulation is provided in Table 1, and the overall experimental schedule is illustrated in Fig. 1.

**Table 1:** Ingredient composition (%) and formulated nutrient profile (g/kg) of the experimental diets.

Item^1^	0–21 d	21-42 d
Ingredient composition(%)	-	-
Corn	10.32	21.27
Wheat middling	15.41	20.00
Wheat bran	-	30.01
Soybean meal	12.63	2.50
Limestone powder	1.52	1.96
Peanut meal	-	2.37
Nucleotide slag	2.00	-
Rice bran	15.81	5.00
Rice noodles	35.21	10.00
Calcium hydrogen phosphate	1.10	0.89
Compound premix^1^	6.00	6.00
Total	100	100
Formulated nutrient profile (g/kg)		
Crude protein	210.00	140.00
Crude fat	20.00	35.00
Crude fiber	50.00	70.00
Crude ash	70.00	100.00
Methionine	4.00	2.80
Phosphorus	6.00	4.50
Sodium chloride	6.00	6.00
Calcium	10.00	10.00
Moisture	140.00	140.00

Ingredient composition is expressed as percentage, whereas formulated nutrient levels are expressed as g/kg on an as-fed basis.

1 Supplied per kilogram of total diet: bentonite, 44.46 g; lysine, 3.24 g; DL-MHA-FA (88%), 0.99 g; threonine, 0.73 g; sodium chloride, 4.40 g; sodium bicarbonate, 2.00 g; sodium sulphate, 2.00 g; herbalife, 0.20 g; choline chloride (60%), 1.00 g; Jin Duowei, 0.53 g; Jin Yvkang, 0.15 g; C-811 enzyme, 0.30 g.

### Determination of growth performance

At the beginning (21 d of age) and end (42 d of age) of the individual test phase, all ducks were weighed after a 12-h fast. Initial body weight (**iBW**) and final body weight (**fBW**) were recorded. Based on traits recorded during the experimental period, including metabolic body weight (**MBW^0.75^**), body weight gain (**BWG**), average daily weight gain (**ADG**), total feed intake (**FI**), and average daily feed intake (**ADFI**), RFI was calculated with a multiple regression model. The calculation formula was as follows:

$$\text{RFI}=\text{ADFI}--\left(a+b_{1}\times \text{MB}W^{0.75}+b_{2}\times \text{ADG}\right)$$

where a denotes the intercept, b_1_ represents the regression coefficient of FI relative to MBW^0.75^, and b_2_ represents the regression coefficient of FI concerning ADG. Sex was not included as a fixed effect in the RFI model or downstream analyses. After RFI calculation, 30 ducks with the lowest RFI values and 30 ducks with the highest RFI values were selected as the LRFI and HRFI groups, respectively, for growth performance analysis. Among these birds, 15 ducks per group were selected for plasma biochemical analysis. For multi-omics analyses, 5 ducks per group were selected from the LRFI and HRFI groups. Cecal contents and hypothalamic tissues were collected from the same individual ducks.

### Collection of experimental samples

At 42 d of age, ducks were fasted for 12 h before sample collection. From the 15 HRFI and 15 LRFI ducks identified based on individual RFI values, approximately 3 mL of blood was collected from the wing vein of each bird into EDTA-K₂ anticoagulant vacuum tubes (5 mL; Chengwu County Medical Products Factory, Shandong, China). Tubes were gently inverted and centrifuged at 3,000 × g for 10 min at 4°C. Plasma was aliquoted into 2-mL cryogenic vials (Corning, NY), snap-frozen in liquid nitrogen, and stored at −80°C for subsequent biochemical analyses.

From these birds, a subset of 5 HRFI and 5 LRFI ducks (n = 5 per group) were euthanized by CO₂ inhalation followed by decapitation. For hypothalamic tissue collection, the skull was opened along the midline to expose the brain. The hypothalamic block was dissected as the region extending from the optic chiasm anteriorly to the mammillary bodies posteriorly, including the tissue surrounding the third ventricle. The tissue was rinsed with pre-cooled PBS (0.1 M, pH 7.4; 4°C) to remove surface blood, placed into sterile 2-mL cryogenic vials, snap-frozen in liquid nitrogen, and stored at −80°C for RNA sequencing.

For cecal sample collection, the abdominal cavity was opened along the midline and the intestines were exposed. Both ceca were excised with sterile scissors and opened longitudinally. Luminal contents adherent to the mucosal surface were scraped, pooled within each individual, homogenized, and divided into two sterile 2-mL cryogenic vials (∼200 mg per vial): one for untargeted LC-MS metabolomics and one for shotgun metagenomic sequencing. All samples were snap-frozen in liquid nitrogen and stored at −80°C until analysis.

### Plasma biochemical and hormonal analysis

Plasma samples from 15 ducks per group were used for biochemical and hormonal analyses. EDTA-K₂ plasma was thawed on ice, gently mixed, and centrifuged at 3,000 × g for 5 min at 4°C to remove residual debris. Plasma concentrations of total cholesterol (**TC**), triglycerides (**TG**), high-density lipoprotein cholesterol (**HDL-C**), low-density lipoprotein cholesterol (**LDL-C**), glucose (**GLU**), and creatine kinase (**CK**) were measured using an automated biochemical analyzer (BS-360E, Mindray, Shenzhen, China) with commercial assay kits (Dirui Industrial Co., Ltd., Changchun, China) according to the manufacturers' instructions. Plasma non-esterified fatty acid (**NEFA**) concentration was determined using a colorimetric assay kit (Elabscience, Wuhan, China; Cat. No. E-BC-K792-M). Plasma concentrations of insulin (**INS**), ghrelin, and leptin (**LEP**) were measured using commercial duck ELISA kits (Jiangsu Enzyme Immunoassay Co., Ltd., Yancheng, China). Plasma growth hormone (**GH**) and corticosterone (**CORT**) were measured using commercial duck ELISA kits (Meiao Biotechnology Co., Ltd., Shanghai, China). Plasma adrenocorticotropic hormone (**ACTH**) was measured using a commercial duck ELISA kit (MLBIO, Shanghai, China). Plasma cholecystokinin (**CCK**) was measured using a commercial duck ELISA kit (Lengton Bioscience Co., Ltd., Shanghai, China). All ELISA procedures were performed according to the manufacturers' instructions, and absorbance was measured using a Synergy H1 microplate reader (BioTek Instruments, Winooski, VT, USA).

### Cecal metagenomic sequencing and data processing

Cecal luminal contents from 5 HRFI ducks and 5 LRFI ducks collected at 42 d of age were used for shotgun metagenomic sequencing. Microbial DNA was extracted using the E.Z.N.A.® Stool DNA Kit (Omega Bio-tek, Norcross, GA, USA). DNA quality and quantity were assessed by agarose gel electrophoresis, NanoDrop spectrophotometry (A260/A280 and A260/A230), and Qubit fluorometric quantification. Metagenomic libraries were constructed using the NEXTflex® Rapid DNA-Seq Kit (Bioo Scientific, Austin, TX, USA) and sequenced on the Illumina NovaSeq platform (Illumina, San Diego, CA, USA) to generate paired-end 150-bp reads, yielding approximately 10 Gb of raw data per sample. Raw reads were quality-filtered to remove adapter sequences, reads containing more than 10% ambiguous bases, and low-quality reads with an average Phred score < 20 in a 10 bp sliding window. Clean reads were aligned to the duck reference genome (Anas platyrhynchos, GCA_003850225.1) using Bowtie2 (v2.5.4), and reads mapped to the host genome were removed. The remaining unmapped reads were retained as microbial reads for downstream analysis. Microbial reads were assembled using MEGAHIT (v1.1.2), and contigs shorter than 500 bp were discarded. Open reading frames were predicted using MetaGeneMark. Predicted genes from all samples were clustered using CD-HIT at 95% sequence identity and 90% coverage to generate a nonredundant gene catalog. Taxonomic annotation was performed by aligning predicted genes against the NCBI nonredundant (**NR**) database using DIAMOND, and taxonomic assignments were obtained based on the best-hit/LCA strategy. Functional annotation of the nonredundant gene catalog was performed using DIAMOND (v0.8.35) against the KEGG, eggNOG, and CAZy databases. For abundance profiling, quality-controlled reads from each sample were mapped back to the nonredundant gene catalog, and gene abundance was normalized based on gene length and mapped read counts for downstream comparisons between groups.

### Cecal untargeted metabolomics analysis

Untargeted metabolomic analysis was performed on cecal contents collected from 5 ducks in the LRFI group and 5 ducks in the HRFI group at 42 d of age. Metabolites were extracted from each sample according to a previously described method (Geng, et al., 2018). Briefly, cecal contents were mixed with extraction solvent, vortexed, and centrifuged, and the resulting supernatants were used for LC-MS analysis. Chromatographic separation was performed using an Agilent 1290 Infinity LC system (Agilent Technologies, Santa Clara, CA, USA) equipped with an ACQUITY UPLC BEH Amide column (100 mm × 2.1 mm, 1.7 μm; Waters, Milford, MA, USA). The autosampler temperature was maintained at 4°C. Mass spectrometric detection was conducted on an AB Sciex TripleTOF 6600 system (AB Sciex, Framingham, MA, USA) in both positive and negative electrospray ionization modes to acquire MS and MS/MS data. To monitor analytical stability, sample injection order was randomized, and one pooled quality control (**QC**) sample was analyzed after every 10 study samples. The QC sample was prepared by pooling equal aliquots from all sample extracts. Multivariate analyses were performed in R using the ropls package (version 1.6.2), including partial least squares discriminant analysis (**PLS-DA**) and orthogonal PLS-DA (**OPLS-DA**). Metabolites with a variable importance in projection (**VIP**) value ≥ 1 in the OPLS-DA model and *P* < 0.05 in a 2-tailed Student’s t-test were considered differential metabolites (**DMs**). Identified metabolites were annotated against the KEGG database for pathway analysis.

### Hypothalamic transcriptomic sequencing and analysis

Hypothalamic tissues from 5 HRFI ducks and 5 LRFI ducks collected at 42 d of age were used for transcriptomic analysis. Total RNA was extracted using TRIzol reagent under RNase-free conditions, and residual genomic DNA was removed by DNase I treatment (Takara, Shiga, Japan). RNA purity was assessed using a NanoDrop 2000 spectrophotometer, and samples with A260/A280 ratios of 1.8 to 2.1 and A260/A230 ≥2.0 were considered acceptable. RNA integrity was further evaluated using an Agilent 2100 Bioanalyzer, and only samples with an RNA integrity number (RIN) ≥ 8.0 and a 28S/18S ratio ≥ 1.5 were used for library preparation. Poly(A)^+^mRNA was enriched from high-quality RNA, and strand-specific cDNA libraries were constructed using a commercial library preparation kit according to the manufacturer’s instructions. Libraries were pooled at equimolar concentrations and sequenced on an Illumina NovaSeq 6000 platform (Illumina, San Diego, CA, USA) to generate 150 bp paired-end reads, yielding at least 6 Gb of raw data per sample. Raw reads were filtered to remove adapter sequences, low-quality reads, and reads containing excessive ambiguous bases. Read quality was assessed using FastQC (Version 0.12.0). Clean reads were aligned to the duck reference genome (Anas platyrhynchos, GCA_003850225.1) using HISAT2 (Version 2.2.1), and gene-level read counts were obtained using featureCounts based on the corresponding gene annotation file. Gene expression levels were also normalized as transcripts per million (**TPM**). Differential expression analysis between HRFI and LRFI ducks was performed using DESeq2 (Version v1.4.2) based on raw read counts. Differentially expressed genes (**DEGs**) were defined as those with |log_2_fold change| > 1 and *P* < 0.05. KEGG pathway enrichment analysis was then performed to identify biological pathways associated with the DEGs.

### Bioinformatics and statistical analysis

Statistical analyses of phenotypic and plasma biochemical data were performed using SPSS software (version 23.0; IBM Corp., Armonk, NY, USA). Data are presented as mean ± SEM. Differences between the HRFI and LRFI groups were evaluated using 2-tailed independent-samples t-tests, with *P* < 0.05 considered statistically significant.

For multi-omics integration, Pearson correlation analysis was used to evaluate pairwise associations among the cecal microbiome, cecal metabolome, and hypothalamic transcriptome. Correlations with *P* < 0.05 were considered significant.

## Results

### Growth performance

Growth performance traits of LRFI and HRFI small-sized meat ducks are presented in Table 2. No significant differences were observed between the 2 groups in iBW (*P* = 0.756), fBW (*P* = 0.923), ADG (*P* = 0.747), or MBW^0.75^ (*P* = 0.926). However, ADFI and FCR were significantly higher in the HRFI group than in the LRFI group (*P* < 0.001).

**Table 2:** Growth performance of small-sized meat ducks in HRFI and LRFI groups (n = 30 per group).

Item^1^	LRFI (mean±SD)	HRFI (mean±SD)	*P-value*
Initial BW (g)	805.50±61.56	793.75±64.80	0.756
Final BW (g)	2069.67±106.76	2076.5 ± 126.76	0.923
ADG (g/d)	60.20±4.24	61.08±4.86	0.747
MBW^0.75 (^g)	306.77±11.85	307.50±14.06	0.926
ADFI (g/d)	131.03±7.88	204.16±13.51	<0.001
FCR (g/g)	2.19±0.19	3.35±0.12	<0.001
RFI (g/d)	−36.92±7.87	35.64±9.99	<0.001

1 HRFI = high residual feed intake; LRFI = low residual feed intake; SEM = standard error of the mean; BW = body weight; ADG = average daily gain; MBW0.75 = metabolic body weight; ADFI = average daily feed intake; FCR = feed conversion ratio; RFI = residual feed intake.

### Biochemical and hormonal indices

The biochemical and hormonal indices of LRFI and HRFI ducks are shown in Table 3 (n = 15 per group). Among the lipid metabolism indices, plasma TG concentration was significantly lower in the LRFI group than in the HRFI group (0.63 ± 0.07 mmol/L vs. 0.76 ± 0.11 mmol/L; *P* = 0.026). No significant differences were observed in TC, HDL-C, or LDL-C between the 2 groups (*P* > 0.05). Among the energy metabolism indices, plasma GLU tended to be lower in the HRFI group (*P* = 0.058), whereas CK activity and NEFA concentration did not differ between groups (*P* > 0.05). For endocrine-related hormones, no significant differences were detected in INS, GH, ACTH, CCK, CORT, ghrelin, or LEP between LRFI and HRFI ducks (*P* > 0.05). Given the limited systemic differences but significant divergence in TG concentration, the cecal microbiome was further analyzed to determine whether microbial composition and functional potential differed between groups.

**Table 3:** Plasma biochemical indices of small-sized meat ducks in low and high RFI groups (n = 15 per group).

Category	Item	LRFI (mean±SD)	HRFI (mean±SD)	*P-value*
Lipid metabolism indices	TC (mmol/L)	4.80±0.31	4.88±0.55	0.763
	TG (mmol/L)	0.63±0.07^a^	0.76±0.11^b^	0.026
	HDL-C (mmol/L)	2.84±0.26	2.85±0.39	0.952
	LDL-C (mmol/L)	1.42±0.24	1.40±0.11	0.886
Energy metabolism indices	GLU (mmol/L)	11.80±0.33	11.34±0.40	0.058
	CK (U/L)	1523.91±282.10	1375.75±300.22	0.396
	NEFA (mmol/L)	0.65±0.13	0.72±0.14	0.383
Endocrine-related hormone indices	INS (uIU/mL)	17.17±2.76	14.88±1.25	0.090
	GH (ng/mL)	4.99±0.37	5.06±0.30	0.709
	ACTH (pg/mL)	26.00±3.61	26.21±3.36	0.922
	CCK (pmol/L)	3.84±0.53	3.62±0.36	0.420
	CORT (ng/ml)	5.60±0.56	5.16±0.17	0.089
	Ghrelin (ng/ml)	85.98±3.81	85.85±3.82	0.955
	LEP (ng/mL)	4.73±0.18	4.72±0.22	0.987

a, b Different superscript letters indicate significant differences between groups, *P* < 0.05.

Plasma parameters were categorized according to their main physiological relevance. TC= Total Cholesterol; TG = Triglyceride; HDL-C = High-Density Lipoprotein Cholesterol; LDL-C = Low-Density Lipoprotein Cholesterol; GLU = Glucose; CK = Creatine Kinase; NEFA = Non-Esterified Fatty Acid; INS = Insulin; GH = Growth Hormone;.

### Cecal metagenomic analysis

**Fig. 2:**
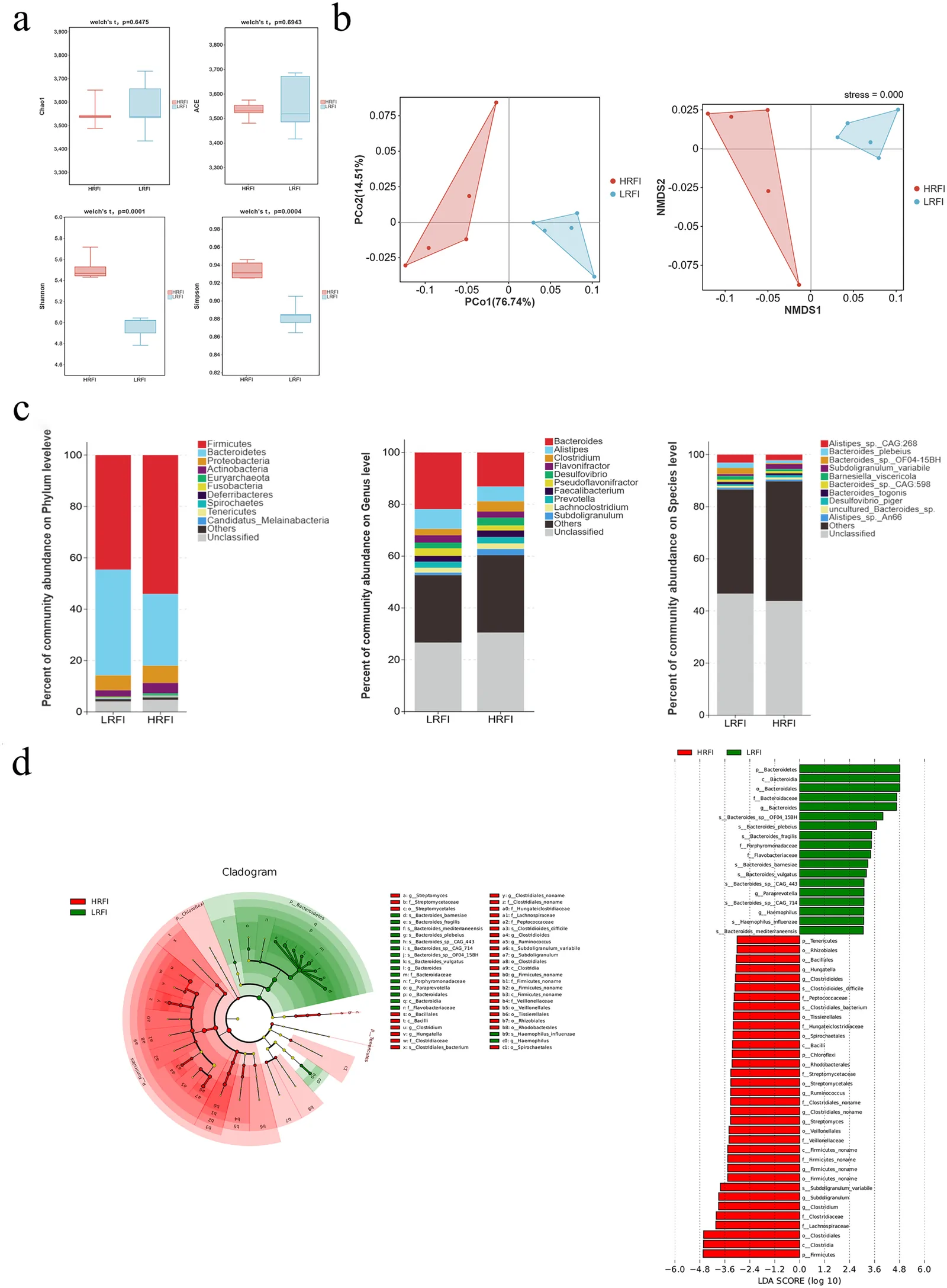
Cecal microbial composition between high and low RFI groups (n = 5 per group). (a) Alpha diversity (Chao1, Ace, Shannon, and Simpson indices) analyzed by t-test. (b) Beta diversity was visualized using principal coordinate analysis (PCoA) and non-metric multidimensional scaling (NMDS) based on Bray-Curtis distance matrices. (c) Cecal microbial composition at the phylum, genus, and species levels. (d) Taxonomic levels and biomarkers identified by LEfSe with LDA scores (*p* < 0.05; LDA > 3.0).

Shotgun metagenomic sequencing of cecal contents (n = 5 per group) generated 772,177,318 high-quality reads after quality control and host read removal, and de novo assembly yielded 3,082,194 contigs (Supplementary Table S1). Alpha-diversity analysis showed no significant differences between LRFI and HRFI ducks in richness, as indicated by the Chao1 (*P* = 0.6475) and ACE (*P* = 0.6943) indices. However, the Shannon and Simpson indices were significantly higher in HRFI than in LRFI ducks (both *P* < 0.001; Fig. 2A), indicating greater microbial diversity and evenness in the HRFI group. PCoA and NMDS analyses showed clear separation between the 2 groups (Fig. 2B).

Metagenomic profiling identified 185 phyla, 200 classes, 507 orders, 1,139 families, 4,248 genera, and 32,835 species in the cecal microbiota of LRFI and HRFI ducks. At the phylum level, the microbiota was dominated by *Firmicutes* and *Bacteroidetes*, followed by *Proteobacteria, Actinobacteria*, and *Euryarchaeota* (Fig. 2C). Compared with LRFI ducks, HRFI ducks showed relatively higher abundance of *Firmicutes* and lower abundance of *Bacteroidetes*. At the genus level, *Bacteroides, Alistipes*, and *Clostridium* were the dominant taxa; *Clostridium* was relatively more abundant in HRFI ducks, whereas *Bacteroides* and *Alistipes* were relatively more abundant in LRFI ducks. At the species level, the predominant taxa included *Alistipes sp. CAG:268, Bacteroides plebeius, Bacteroides sp. OF04-15BH, Subdoligranulum variabile*, and *Barnesiella viscericola* (Fig. 2C). LEfSe analysis identified multiple differential taxa between groups (*P* < 0.05, LDA > 3.0; Fig. 2D). Notably, *Bacteroides*, including *Bacteroides sp. OF04-15BH* and *Bacteroides plebeius*, was enriched in LRFI ducks, whereas *Subdoligranulum variabile* was enriched in HRFI ducks, suggesting that these taxa may be associated with divergence in feed efficiency.

### Functional profile of the cecal microbiota

**Fig. 3:**
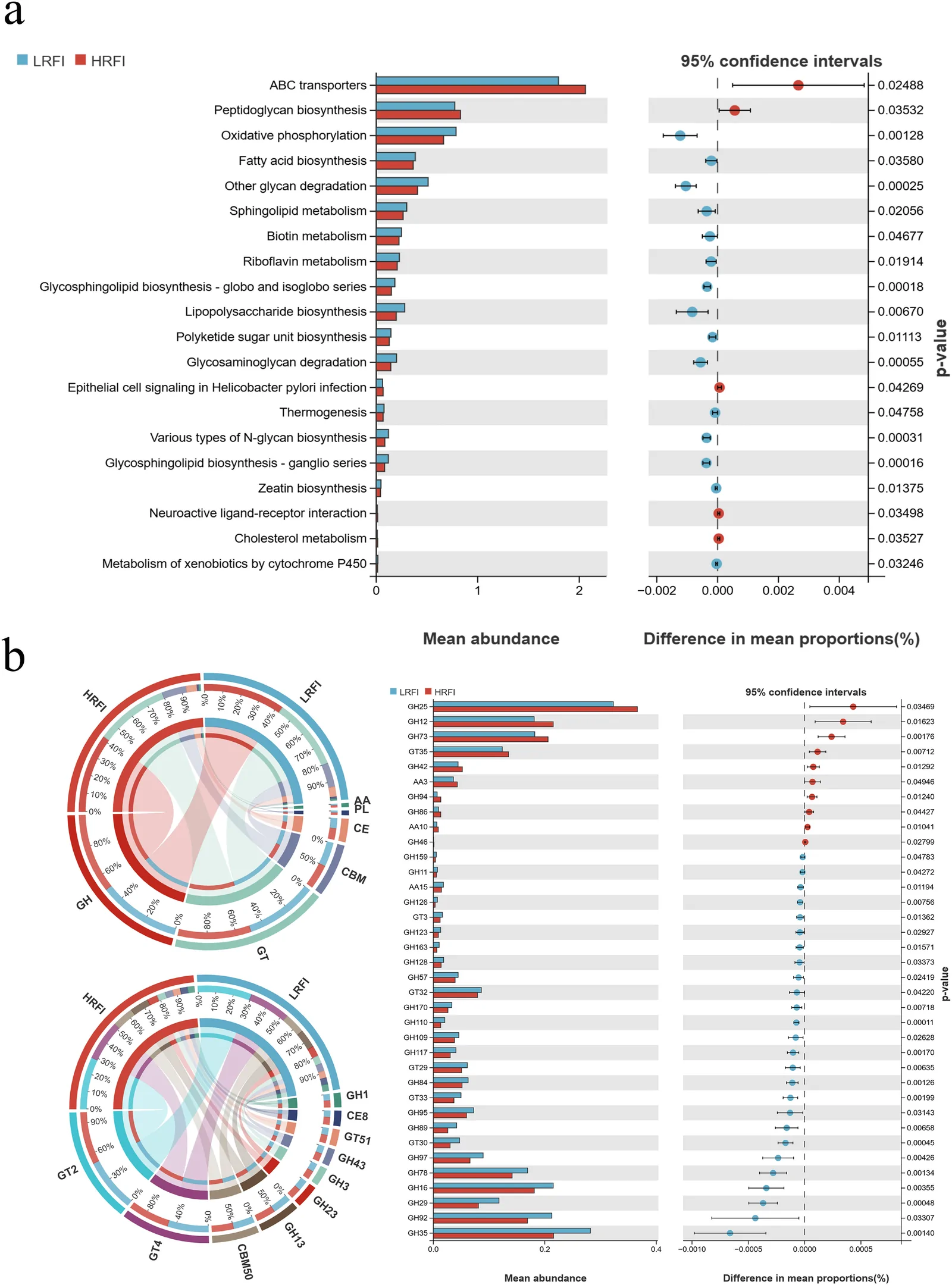
Functional profiles of cecal microbiota between HRFI and LRFI groups. (a) Differential KEGG functional analysis of cecal microbiota in high and low RFI small-sized meat ducks (TOP 20). (b) CAZy functional analysis of cecal microbial enzymes and screening of significantly differentially abundant microbial enzymes.

The functional potential of the cecal microbiome was evaluated based on KEGG pathway annotation and CAZyme profiling. As shown in Fig. 3A, multiple KEGG pathways differed in relative abundance between LRFI and HRFI ducks. Compared with the LRFI group, the HRFI group showed lower relative abundances of genes involved in oxidative phosphorylation, other glycan degradation, glycosaminoglycan degradation, thermogenesis, fatty acid biosynthesis, sphingolipid metabolism, glycosphingolipid biosynthesis, biotin metabolism, and riboflavin metabolism (Welch's t-test, *P* < 0.05). In contrast, genes associated with ABC transporters, cholesterol metabolism, peptidoglycan biosynthesis, and neuroactive ligand–receptor interaction were more abundant in the HRFI group (*P* < 0.05).

A total of 376 CAZyme families were identified, including 143 glycoside hydrolases (**GH**), 98 glycosyltransferases (**GT**), 74 carbohydrate-binding modules (**CBM**), 18 carbohydrate esterases (**CE**), 31 polysaccharide lyases (**PL**), and 12 auxiliary activities (**AA**) (Fig. 3B). Among these, 36 CAZyme families differed significantly between groups (Welch’s t-test, *P* < 0.05). The HRFI group showed higher relative abundances of several GH and AA families, including GH25, GH12, GH73, GH42, GH94, GH86, GH46, AA3, and AA10, whereas the LRFI group had higher relative abundances of GH35, GH92, GH29, GH16, GH78, GH97, GH30, GH89, GH95, GH84, GT30, GT33, GT29, GT32, and GT3. These results suggest differences in the functional potential of the cecal microbiome between LRFI and HRFI ducks. Untargeted metabolomic profiling of cecal contents was then performed to further characterize metabolic differences between groups.

### Identification of differential cecal metabolites

**Fig. 4:**
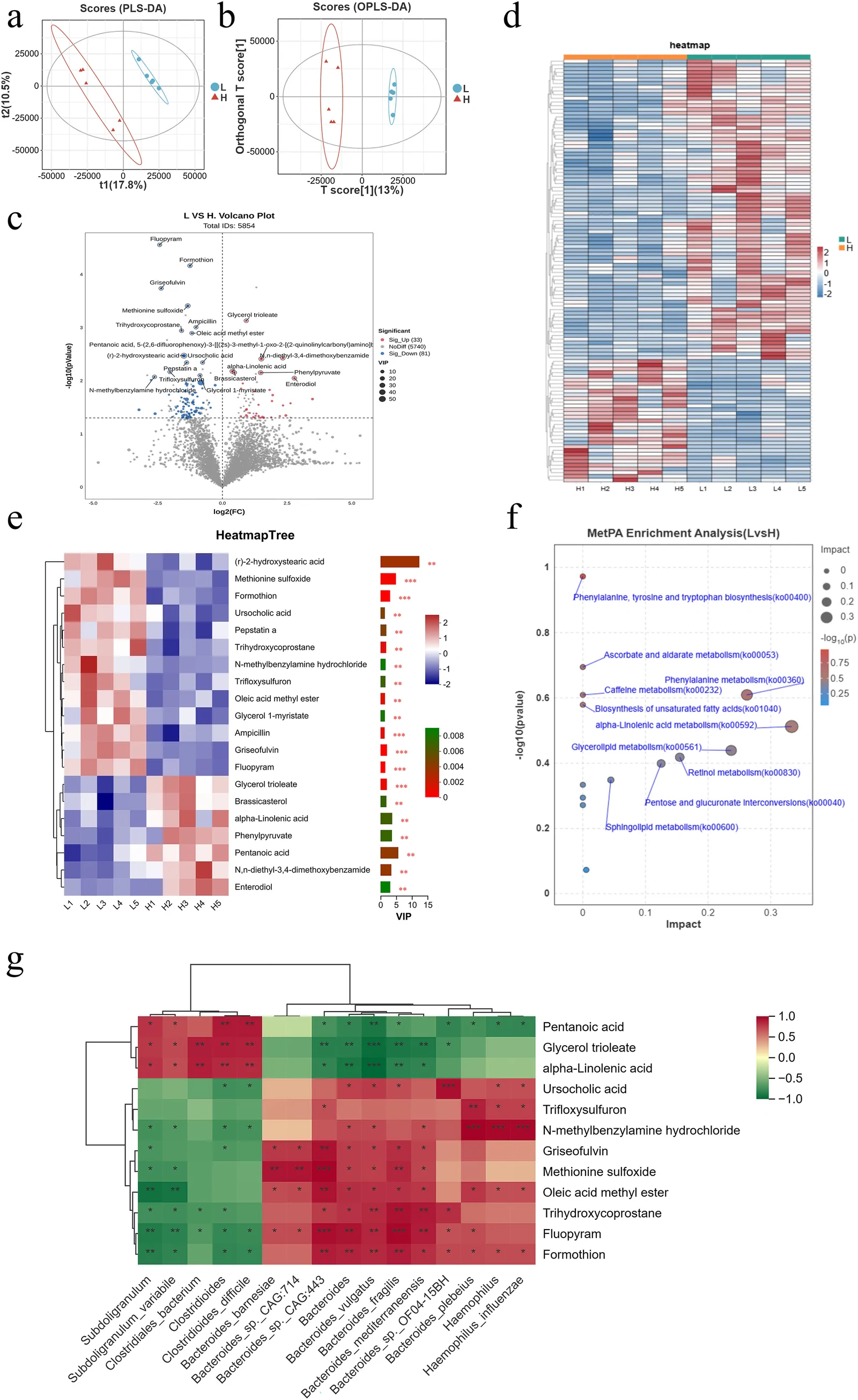
Untargeted metabolomic analysis of cecal contents in LRFI and HRFI groups (n = 5 per group). (a) PLS-DA analysis (combination of positive and negative ions). (b) OPLS-DA analysis (combination of positive and negative ions). (c) Volcano plot of DMs. (d) Expression of DMs in the clustered heatmap. (e) VIP value analysis of the top 20 differential metabolites. (f) MetPA pathway enrichment analysis. (g) Correlation analysis between cecal microbiota and DMs (*p < 0.05, ** *p* < 0.01, *** *p* < 0.001).

Untargeted LC-MS analysis of cecal contents from LRFI and HRFI ducks detected 5,855 metabolic features. PLS-DA and OPLS-DA score plots showed a clear separation between the 2 groups (Fig. 4A-B). Using the criteria of variable importance in projection (**VIP**) ≥ 1 and *P* < 0.05, 114 DMs were identified between LRFI and HRFI ducks. Compared with the LRFI group, the HRFI group had 33 increased and 81 decreased metabolites (Fig. 4C-D). The main discriminatory metabolites included methionine sulfoxide, glycerol trioleate, oleic acid methyl ester, (R)-2-hydroxystearic acid, pentanoic acid, ursocholic acid, alpha-linolenic acid, phenylpyruvate, and glycerol 1-myristate (Fig. 4E). Pathway analysis identified 14 KEGG pathways, and the DMs were mainly involved in phenylalanine, tyrosine and tryptophan biosynthesis (ko00400), phenylalanine metabolism (ko00360), biosynthesis of unsaturated fatty acids (ko01040), alpha-linolenic acid metabolism (ko00592), glycerolipid metabolism (ko00561), and sphingolipid metabolism (ko00600) (Fig. 4F).

### Microbe–metabolite associations in the cecum

Pairwise associations between selected DMs and discriminative microbial taxa were evaluated using Pearson correlation analysis (Fig. 4G). *Subdoligranulum variabile* was positively correlated with pentanoic acid, glycerol trioleate, and alpha-linolenic acid, and negatively correlated with methionine sulfoxide and oleic acid methyl ester. *Clostridioides difficile* was also positively correlated with pentanoic acid, glycerol trioleate, and alpha-linolenic acid. Several *Bacteroides* species were significantly associated with multiple metabolites. Specifically, *Bacteroides sp. OF04-15BH* was negatively correlated with pentanoic acid and glycerol trioleate, but positively correlated with ursocholic acid, whereas *Bacteroides plebeius* was negatively correlated with pentanoic acid and positively correlated with oleic acid methyl ester (all *P* < 0.05). Based on these associations, hypothalamic transcriptomes from the same individuals were further analyzed to investigate potential central molecular differences between groups.

### Hypothalamic transcriptome analysis

**Fig. 5:**
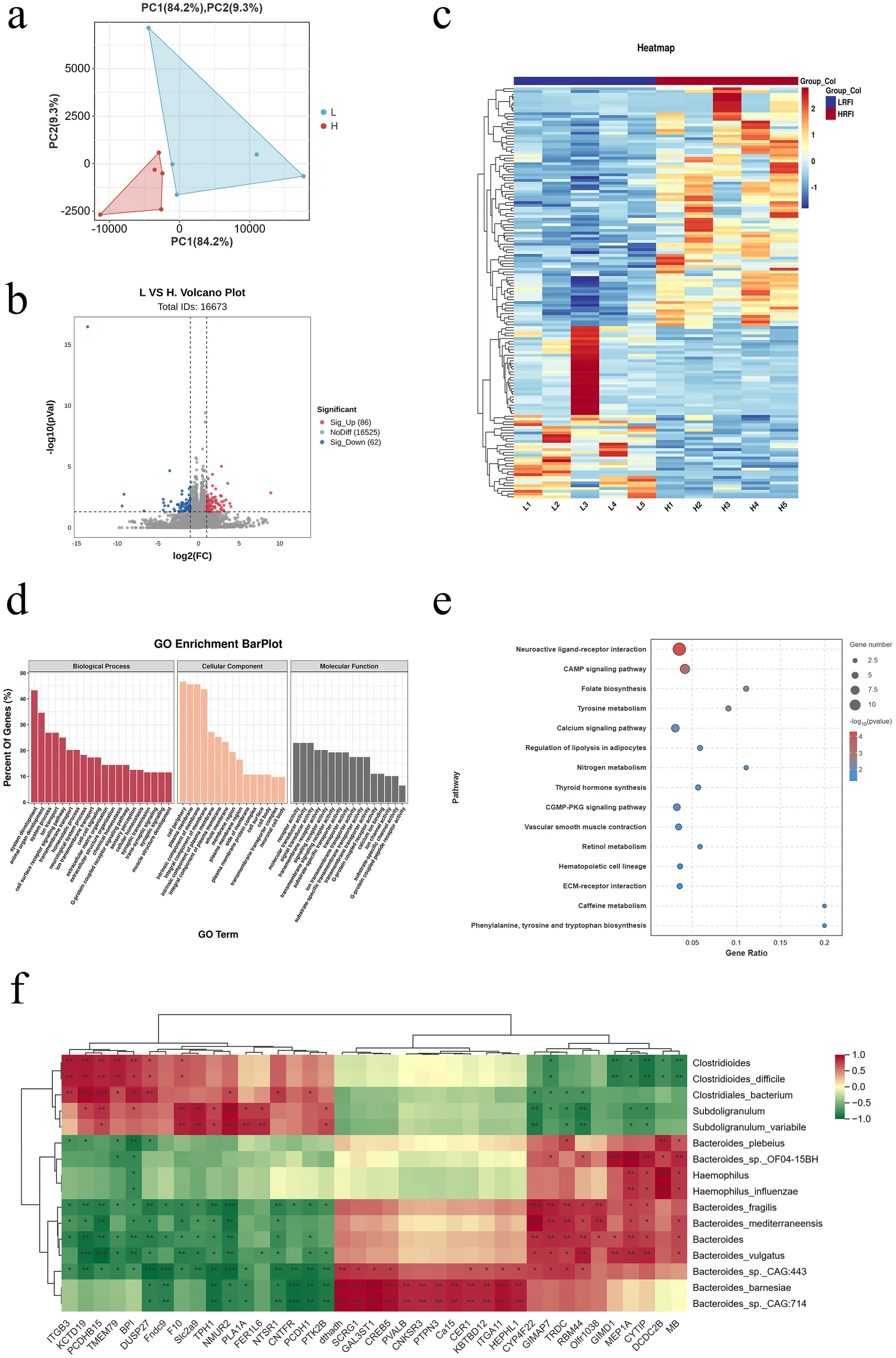
RNA-seq data analysis of the hypothalamus in LRFI and HRFI groups (n = 5 per group). (a) Principal component analysis plot. (b) Gene volcano plot. (c) Expression of DEGs in the cluster heatmap. (d) GO annotation analysis of DEGs. (e) KEGG pathway enrichment analysis of DEGs. (f) Correlation heatmap between DEGs and signature microorganisms (*p < 0.05, ** *p* < 0.01, *** *p* < 0.001).

RNA-seq analysis of hypothalamic tissue was performed in LRFI and HRFI ducks. After quality filtering, each library contained 46.4 to 56.9 million clean reads, of which approximately 90.0% mapped to the duck reference genome. Q20 and Q30 values exceeded 96% and 91%, respectively, and GC content ranged from 46% to 47%, indicating that the sequencing data were of high quality and suitable for downstream analysis (Supplementary Table S2). PCA based on gene expression profiles showed clear separation between LRFI and HRFI samples (Fig. 5A). Using the criteria of |log_2_fold change| ≥ 1 and *P* < 0.05, differential expression analysis identified 148 DEGs among 16,673 detected genes. Compared with the LRFI group, 86 genes were upregulated and 62 genes were downregulated in the HRFI group (Fig. 5B-C).

GO enrichment analysis of the 148 DEGs identified 688 significantly enriched terms (*P* < 0.05; Fig. 5D). In the Biological Process category, DEGs were mainly enriched in system development, organ development, muscle structure development, and cell-cell or receptor-mediated signaling. In the Cellular Component category, enriched terms included cell periphery, plasma membrane, intrinsic component of membrane, and transmembrane transporter complex. In the Molecular Function category, enriched terms were mainly related to receptor activity, signaling receptor activity, G protein-coupled receptor activity, and signal transducer activity. KEGG pathway analysis identified 24 significantly enriched pathways (*P* < 0.05; Fig.5E). After excluding human disease-related pathways, the enriched pathways were mainly involved in environmental information processing, including neuroactive ligand-receptor interaction, cAMP signaling pathway, calcium signaling pathway, and cGMP-PKG signaling pathway; metabolism, including folate biosynthesis, tyrosine metabolism, nitrogen metabolism, retinol metabolism, and phenylalanine / tyrosine / tryptophan biosynthesis; and organismal systems, including regulation of lipolysis in adipocytes, thyroid hormone synthesis, and vascular smooth muscle contraction. Representative DEGs included signaling- and receptor-related genes (*MB, TRDC, ITGA11, PTK2B, NMUR2, ITGB3, CREB5, NTSR1, TSHR*) and metabolism-related genes (*TH, RETSAT, NAT1, TPH1, CA7, CY P26B1*).

### Associations between hypothalamic DEGs and cecal taxa

Pearson correlation analysis was performed between representative hypothalamic DEGs and discriminative cecal taxa (Fig.5F). *Clostridioides*, including *Clostridioides difficile* and *Clostridiales bacterium*, was positively correlated with *ITGB3, KCT D19, PCDHB15, TMEM79, BPI*, and *DUSP27. Subdoligranulum* and *Subdoligranulum variabile* were positively correlated with *F10, SLC2A9, TPH1, NMUR2, PLA1A, FER1L6*, and *PTK2B*, but negatively correlated with *CYP4F22, TRDC*, and *MEP1A*. Multiple *Bacteroides* species were also associated with hypothalamic gene expression. *Bacteroides plebeius* was negatively correlated with *ITGB3, KCTD19, TMEM79, BPI*, and *DUSP27*, but positively correlated with *TRDC, DCDC2B*, and *MB. Bacteroides sp. OF04-15BH* was positively correlated with *GIMD1, MEP1A, CYTIP, DCDC2B*, and *MB*.

### Multi-omics integration of cecal microbiome, metabolome, and hypothalamic transcriptome

**Fig. 6:**
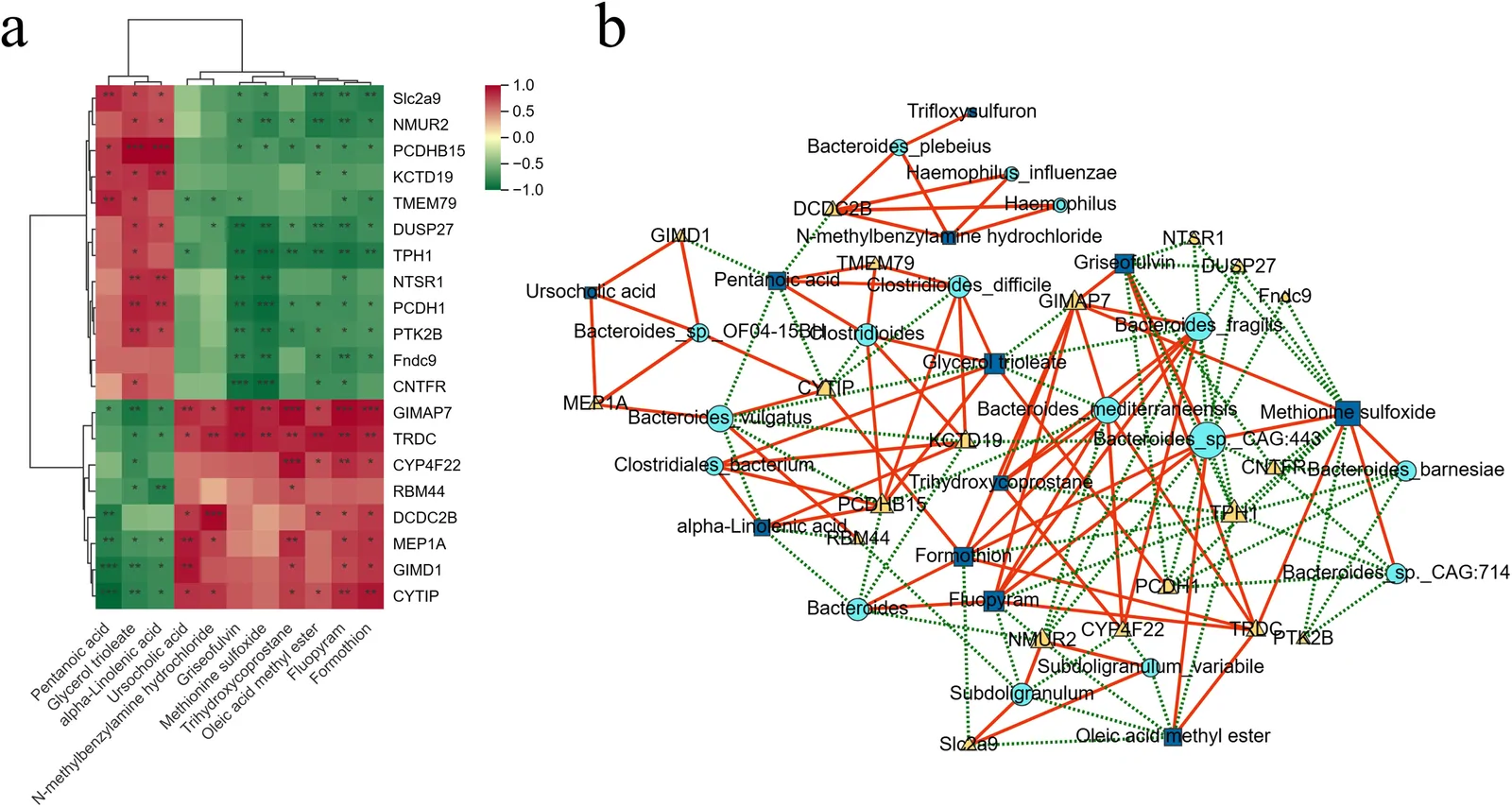
Integrated correlation analysis of discriminative cecal taxa, DMs, and hypothalamic DEGs in small-sized meat ducks divergent for RFI. (a) Heatmap showing Pearson correlation coefficients between DMs and representative hypothalamic DEGs (*p < 0.05, ** *p* < 0.01, *** *p* < 0.001). (b) Integrated multi-omics correlation network showing the associations among discriminative cecal taxa, DMs, and representative hypothalamic DEGs. Solid red lines indicate positive correlations, whereas green dashed lines indicate negative correlations.

To explore the potential linkages among the cecal microbiome, cecal metabolome, and hypothalamic transcriptome, Pearson correlation analysis was performed between DMs and representative hypothalamic DEGs (Fig. 6A). Hypothalamic genes upregulated in HRFI ducks, including *SLC2A9, NMUR2, PCDHB15, KCTD19, TMEM79, DUSP27, TPH1, NTSR1, PCDH1*, and *PTK2B*, were positively correlated with glycerol trioleate and alpha-linolenic acid, but negatively correlated with methionine sulfoxide, oleic acid methyl ester, and trihydroxycoprostane. Conversely, hypothalamic genes upregulated in LRFI ducks, including *GIMAP7, TRDC, MEP1A, GIMD1*, and *CYTIP*, were positively correlated with ursocholic acid, oleic acid methyl ester, and trihydroxycoprostane, but negatively correlated with pentanoic acid, glycerol trioleate, and alpha-Linolenic acid.

An integrated multi-omics correlation network was further constructed to visualize the tripartite associations among discriminative cecal taxa, DMs, and hypothalamic DEGs (Fig. 6B). Several taxa, including *Bacteroides sp. OF04-15BH, Bacteroides plebeius, Bacteroides fragilis, Bacteroides mediterraneensis, Clostridioides difficile, Clostridiales_bacterium*, and *Subdoligranulum variabile*, occupied relatively central positions in the network and were connected with multiple metabolites and hypothalamic DEGs. Among the metabolites, pentanoic acid, alpha-Linolenic acid, ursocholic acid, glycerol trioleate, trihydroxycoprostane, and oleic acid methyl ester served as major connecting nodes linking cecal microbial taxa with hypothalamic gene expression patterns. For example, ursocholic acid was positively associated with *Bacteroides sp. OF04-15BH* and *MEP1A*, whereas pentanoic acid and glycerol trioleate were connected with *Clostridioides difficile* and genes such as *TMEM79, GIMD1, CYTIP, PCDH1*, and *PCDHB15*. In addition, oleic acid methyl ester was linked with *Subdoligranulum variabile* and hypothalamic genes including *TPH1, TRDC, NMUR2*, and *SLC2A9*. Notably, both positive and negative correlations were observed within the network, indicating complex association patterns among the cecal microbiota, luminal metabolites, and hypothalamic transcriptomic features in ducks with divergent RFI.

## Discussion

RFI is a complex feed-efficiency trait influenced by feed intake behavior, nutrient digestion, intestinal microbial activity, host metabolism, immune responses, and neuroendocrine regulation. Recent poultry studies have shown that divergent RFI phenotypes are not only associated with differences in growth and feed intake, but also with alterations in gut microbiota, metabolite profiles, and host gene expression. In ducks, previous studies have linked RFI variation with intestinal microbiome and metabolome changes, gut-brain axis-related gene expression, and host–microbiota interactions (Bai, et al., 2024). Therefore, the present study was interpreted within this broader biological framework by integrating cecal metagenomics, cecal metabolomics, and hypothalamic transcriptomics. Selection for low RFI has been proposed as a practical strategy to improve feed efficiency in meat ducks without compromising growth or carcass traits (Bai, et al., 2022a; Chang, et al., 2024). Consistent with this, LRFI ducks in the present study consumed less feed and exhibited a lower FCR than HRFI ducks, whereas iBW, fBW, MBW^0.75^, and ADG did not differ between the 2 groups, indicating that the divergence in RFI was driven by differences in feed intake and nutrient utilization rather than growth potential. This decoupling between intake and gain supports the use of RFI as a selection criterion that captures individual variation in metabolic efficiency independent of growth rate, as also reported in cattle and sheep (Sainz, et al., 2024; Zhang, et al., 2023). At the systemic level, plasma TG concentrations were significantly lower in LRFI ducks, consistent with the observation that feed-efficient poultry tend to exhibit reduced lipid mobilization (Zhang, et al., 2026). In poultry, a lower plasma TG level in the context of comparable growth performance suggests that LRFI ducks may partition less dietary energy toward lipid storage, thereby contributing to their superior feed efficiency. CK activity did not differ significantly between the 2 groups, suggesting that the metabolic distinction between LRFI and HRFI ducks is more closely related to lipid metabolism than to muscle energy turnover. These phenotypic and biochemical observations prompted us to investigate the underlying biological mechanisms through cecal microbiome, metabolome, and hypothalamic transcriptome profiling.

Gut microbiota has increasingly been recognized as an important biological factor associated with feed efficiency in poultry. Previous studies in chickens and ducks have shown that differences in RFI or digestive efficiency are accompanied by changes in intestinal microbial diversity, microbial composition, and functional potential. Beta diversity analyses revealed clear separation between LRFI and HRFI cecal communities, supporting an RFI-associated restructuring of the microbiota. Alpha diversity indices reflecting richness (Chao1 and ACE) did not differ between the 2 groups; however, Shannon and Simpson indices were significantly higher in HRFI ducks, indicating greater evenness in the less efficient group. At the phylum level, LRFI ducks exhibited a decrease in *Firmicutes* and an increase in *Bacteroidetes*. A higher *Firmicutes*-to-*Bacteroidetes* ratio has frequently been associated with increased energy harvest and adiposity (Wang, et al., 2025), although this relationship is not universal. Members of the *Bacteroidetes*, particularly *Bacteroides*, are well-recognized degraders of complex polysaccharides and may contribute to more efficient nutrient extraction from fibrous dietary components (Li, et al., 2024). At the genus level, *Bacteroides* and *Alistipes* were more abundant in LRFI ducks, whereas *Clostridium* was more prevalent in HRFI ducks. Some *Clostridium* members have been linked to pro-inflammatory activity in the gut (Xu, et al., 2025a; Zhao, et al., 2024), so their reduced abundance in LRFI ducks may reflect a more metabolically favorable intestinal environment, although species-level resolution will be required to confirm this. LEfSe analysis further identified *Bacteroides sp. OF04-15BH* and *Bacteroides plebeius* as characteristic of LRFI, and *Subdoligranulum variabile* as characteristic of HRFI. Functional prediction analysis revealed that pathways related to energy metabolism (oxidative phosphorylation and thermogenesis), lipid metabolism (fatty acid biosynthesis), and cofactor/vitamin metabolism (biotin and riboflavin) were enriched in LRFI ducks, suggesting that the LRFI-associated microbiota possesses a greater predicted capacity for energy conversion and cofactor synthesis. Analysis of CAZymes further supported this functional divergence: a broader range of GH and GT families were more abundant in LRFI ducks. In contrast, ABC transporters and cholesterol metabolism were more prevalent in HRFI ducks, which may indicate altered substrate transport and lipid handling in the less efficient cecum. The enrichment of the neuroactive ligand–receptor interaction pathway in HRFI ducks is noteworthy, as certain gut microbes can produce neuroactive compounds, including gamma-aminobutyric acid and serotonin precursors, that may participate in microbiota–gut–brain communication (Khan, et al., 2025; Qu, et al., 2024).

Metabolic regulation represents another important layer linking intestinal microbial activity with feed efficiency. Previous duck RFI studies using microbiome and metabolome analyses reported that divergent RFI phenotypes were associated with differential metabolites and pathways related to amino acid metabolism, vitamin metabolism, lipid metabolism, and membrane transport (Guo, et al., 2025). These findings suggest that metabolite-mediated host–microbe interactions may contribute to individual differences in feed utilization. Metabolomic profiling of the cecal contents revealed distinct metabolic landscapes between the 2 RFI groups. Glycerol trioleate, pentanoic acid, and alpha-linolenic acid were elevated in HRFI ducks, whereas oleic acid methyl ester and bile acid-related metabolites, such as ursocholic acid, were enriched in LRFI ducks. It is worth noting that pentanoic acid is a short-chain fatty acid that has been implicated in gut barrier integrity and gut–brain signaling (Hao, et al., 2023), rather than a lipid storage molecule. Its elevation in HRFI ducks may reflect differences in microbial fermentation patterns. In contrast, the enrichment of ursocholic acid in LRFI ducks is of particular interest, as bile acids are important signaling molecules that regulate lipid absorption, energy expenditure, and host–microbe interactions (Chan, et al., 2026; Mohanty, et al., 2024). DMs were enriched in pathways including phenylalanine, tyrosine, and tryptophan biosynthesis; biosynthesis of unsaturated fatty acids; alpha-linolenic acid metabolism; and sphingolipid metabolism. These pathways are closely associated with nutrient and energy supply, membrane lipid remodeling, and the generation of signaling precursors (Qin, et al., 2024; Tan, et al., 2023; Xu, et al., 2025b). Pearson correlation analysis linked these metabolic shifts to specific microbial taxa: *Subdoligranulum variabile* and *Clostridioides difficile*, enriched in HRFI ducks, were positively correlated with glycerol trioleate and alpha-linolenic acid, whereas *Bacteroides sp. OF04-15BH* was positively correlated with ursocholic acid but negatively correlated with pentanoic acid and glycerol trioleate. These correlations suggest that the divergent cecal metabolite profiles are, at least in part, shaped by compositional differences in the cecal microbiota.

The hypothalamus is a central integrator of peripheral metabolic signals and a key regulator of feeding behavior and energy homeostasis (Chen, et al., 2025a). We identified 148 DEGs between LRFI and HRFI ducks, which were enriched in GO terms related to G protein-coupled receptor signaling, transmembrane transport, and signal transducer activity, and in KEGG pathways including neuroactive ligand–receptor interaction, cAMP signaling, and tyrosine metabolism. These findings suggest that the hypothalamus in LRFI and HRFI ducks undergoes transcriptional remodeling in pathways linked to sensing and integrating peripheral signals. Correlation analysis revealed candidate microbiota–brain associations. *Subdoligranulum variabile*, enriched in HRFI ducks, was positively correlated with *NMUR2, TPH1*, and *PTK2B. NMUR2* encodes the neuromedin U receptor 2, a key mediator of the anorexigenic effects of neuromedin U in the hypothalamus; central administration of NMU suppresses food intake in both mammals and poultry (Botticelli, et al., 2023; Ghashghayi, et al., 2024). *TPH1* encodes tryptophan hydroxylase 1, primarily responsible for peripheral serotonin synthesis, although its expression has also been detected in discrete brain regions (Nonogaki and Kaji, 2025; Sbrini, et al., 2022). Given the established role of serotonin in appetite regulation, the positive correlation between *Subdoligranulum variabile* and *TPH1* expression warrants further investigation. In contrast, several LRFI-associated *Bacteroides* species exhibited positive correlations with *DCDC2B* and *MEP1A*, the latter encoding a metalloprotease linked to epithelial barrier remodeling and inflammatory tone (Grainger, et al., 2021). These patterns suggest that LRFI- and HRFI-associated taxa are connected to distinct hypothalamic transcriptional modules.

To further explore the tripartite relationships among cecal microbiota, metabolites, and hypothalamic gene expression, an integrated association network was constructed. The network confirmed and extended the pairwise correlations described above, revealing coordinated multi-omics association patterns. For example, ursocholic acid was positively associated with *Bacteroides sp. OF04-15BH* and *MEP1A*, suggesting a potential link between LRFI-enriched *Bacteroides* lineages, bile acid signaling, and hypothalamic gene programs related to barrier function. *Bacteroides* species are known to possess bile salt hydrolase activity and can actively modify the host bile acid pool (Han, et al., 2025), which may in turn serve as a signaling axis to the host. In contrast, pentanoic acid and glycerol trioleate were connected with *Clostridioides difficile* and hypothalamic genes including *TMEM79, GIMD1, CYTIP*, and *PCDHB15*, several of which are involved in transmembrane signaling and cell adhesion. The co-occurrence of these HRFI-associated metabolites with genes related to intercellular communication raises the possibility that microbially derived lipid and SCFA signals may contribute to altered neuroendocrine tone in HRFI ducks. In addition, oleic acid methyl ester was linked with *Subdoligranulum variabile* and hypothalamic genes including *TPH1, TRDC, NMUR2*, and *SLC2A9. SLC2A9* encodes a urate transporter with additional roles in glucose handling, and its co-association with appetite-related genes such as *NMUR2* and *TPH1* points to a broader nutrient-sensing module in the HRFI hypothalamus. While these associations are correlative and do not establish causality, they generate testable hypotheses regarding microbiota-mediated modulation of hypothalamic function and feeding behavior.

Compared with previous poultry RFI studies that mainly focused on growth performance, gut microbiota, metabolomics, transcriptomics, or two-omics associations, this work provides one of the first integrated views of cecal microbial composition and function, cecal metabolites, and hypothalamic gene expression in ducks stratified by RFI. By combining shotgun metagenomics, untargeted metabolomics, and RNA sequencing, the present study extends previous duck RFI research and provides a broader framework for understanding potential microbiota-metabolite-hypothalamus interactions associated with feed efficiency. However, several limitations must be acknowledged. First, no gnotobiotic, colonization, or dietary manipulation experiments were performed, so causality cannot be assigned to any of the associations identified. Future studies should employ germ-free or microbiota-transplant models, or targeted colonization with candidate taxa such as *Bacteroides* or *Subdoligranulum*, to determine whether specific microbial lineages can modulate hypothalamic signaling and feed efficiency. Second, the relatively small sample size for multi-omics integration is another limitation of this study. Although nominally significant correlations were observed among cecal microbiota, metabolites, and hypothalamic genes, most associations did not remain significant after FDR correction. Therefore, these findings should be interpreted as exploratory and hypothesis-generating, and further validation in larger cohorts is needed. Third, one limitation of this study is that mixed-sex ducks were used, and sex was not included as a fixed effect in the downstream analyses. Although RFI was calculated based on individual feed intake and growth performance, potential sex-related effects cannot be completely excluded. Future studies with larger sample sizes and sex-stratified analyses are warranted. Nevertheless, the coordinated patterns identified here provide a framework for developing microbiota-informed nutritional strategies and marker-assisted selection aimed at improving feed efficiency in meat-type ducks.

## Author contributions

Hao Bai and Guobin Chang conceived of and designed the study. Dandan Geng, Yifan Ding, Yong Jiang, and Zhixiu Wang conducted the tests and engaged in data gathering. Dandan Geng conducted data analysis and authored the manuscript. Guohong Chen and Guobin Chang amended the manuscript. All authors participated in and endorsed the submitted work.

## Disclosures

No potential conflict of interest was reported by the authors.

## Data Availability

None of the data were deposited in an official repository. Data is available upon request.

## Appendix Supplementary materials

Image, application 1

Image, application 2

## Footnotes

Supplementary material associated with this article can be found, in the online version, at doi:10.1016/j.psj.2026.107310.
